# Three-Dimensional Carbon Allotropes Comprising Phenyl Rings and Acetylenic Chains in *sp*+*sp*^2^ Hybrid Networks

**DOI:** 10.1038/srep24665

**Published:** 2016-04-18

**Authors:** Jian-Tao Wang, Changfeng Chen, Han-Dong Li, Hiroshi Mizuseki, Yoshiyuki Kawazoe

**Affiliations:** 1 Beijing National Laboratory for Condensed Matter Physics, Institute of Physics, Chinese Academy of Sciences, Beijing 100190, China; 2 Department of Physics and High Pressure Science and Engineering Center, University of Nevada, Las Vegas, Nevada 89154, USA; 3 State Key Laboratory of Environmental Criteria and Risk Assessment, Chinese Research Academy of Environmental Sciences, Beijing 100012, China; 4 Computational Science Research Center, Korea Institute of Science and Technology (KIST), Hwarangno 14-gil 5, Seongbuk-gu, Seoul 02792, Republic of Korea; 5 New Industry Creation Hatchery Center, Tohoku University, Sendai 980-8579, Japan; 6 Institute of Thermophysics, Siberian Branch of Russian Academy of Sciences, Novosibirsk 630090, Russia

## Abstract

We here identify by *ab initio* calculations a new type of three-dimensional (3D) carbon allotropes that consist of phenyl rings connected by linear acetylenic chains in *sp*+*sp*^2^ bonding networks. These structures are constructed by inserting acetylenic or diacetylenic bonds into an all *sp*^2^-hybridized rhombohedral polybenzene lattice, and the resulting 3D phenylacetylene and phenyldiacetylene nets comprise a 12-atom and 18-atom rhombohedral primitive unit cells in the 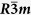 symmetry, which are characterized as the 3D chiral crystalline modification of 2D graphyne and graphdiyne, respectively. Simulated phonon spectra reveal that these structures are dynamically stable. Electronic band calculations indicate that phenylacetylene is metallic, while phenyldiacetylene is a semiconductor with an indirect band gap of 0.58 eV. The present results establish a new type of carbon phases and offer insights into their outstanding structural and electronic properties.

The valence electrons of carbon atom are capable of forming *sp*^3^-, *sp*^2^- and *sp*-hybridized states that support four basic types of single, double, triple, and aromatic carbon-carbon bonds^1^,^2^,^3^,^4^. These different bonding states in various compounds have a profound impact on a wide range of properties of carbon-based materials. At ambient conditions, graphite, which is structurally related to polycyclic benzenoid aromatic hydrocarbon, is the thermodynamically most stable carbon configuration. The polycyclic carbon atoms form a two-dimensional (2D) benzenoid *sp*^2^ bonding network with a bond angle of 120° and a bond length of 0.142 nm. Diamond, which is related to polycyclic saturated hydrocarbon, is the second most stable allotrope of carbon with all the carbon atoms in a methane-like tetrahedral *sp*^3^ bonding state with a bond angle of 109.5° and a bond length of 0.154 nm as in alkanes, forming a very rigid three-dimensional (3D) carbon network. Linear carbyne, which is related to polyyne-like unsaturated hydrocarbon with alternating single and triple carbon-carbon bonds rather than a cumulene structure, forming the simplest one-dimensional (1D) carbon chain, and it has been recently synthesized^4^, despite its rather high energy of about 1 eV per atom above that of graphite.

Since the discovery of fullerenes^5^, nanotubes^6^, and graphene^7^, considerable theoretical and experimental efforts have been made to search and assess new potential carbon allotropes^8^,^9^,^10^,^11^,^12^,^13^,^14^,^15^,^16^,^17^,^18^,^19^,^20^. Intriguing among them are the so-called graphyne and graphdiyne^21^,^22^,^23^,^24^, which are constructed by replacing one-third of the C–C bonds in graphene sheet with acetylenic (–C≡C–) or diacetylenic (–C≡C–C≡C–) linkages. Analogously, graphyne nanotubes^25^ and fullereneynes^26^ are also proposed theoretically. Thus far, large-scale graphyne and graphdiyne films composed of *sp*+*sp*^2^ hybrid network^27^,^28^,^29^ have been successfully synthesized under laboratory conditions. Motivated by *sp*+*sp*^2^-hybridized 2D carbon allotropes, an *sp*+*sp*^3^-hybridized 3D diamondyne was suggested by inserting triple yne-bonds into all the C–C bonds in cubic diamond^30^,^31^,^32^. In addition, a new material termed porous aromatic frameworks (PAFs) via inserting rigid phenyl rings into all the C–C bonds of diamond was reported^33^,^34^,^35^. These studies open a new approach to constructing 3D covalent carbon network structures.

In this work we report *ab initio* total-energy and phonon calculations^36^,^37^,^38^,^39^,^40^,^41^ that predict a new type of 3D carbon phases in 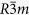 (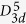) symmetry constructed by inserting linear acetylenic chains into an *sp*^2^-hybridized rh6 polybenzene lattice^3^. The resulting 3D network structures of phenylacetylene and phenyldiacetylene consist of benzene rings bonding together with acetylene or diacetylene, which topologically correspond to 2D graphyne and graphdiyne, respectively. They are energetically more favorable than the *sp*-hybridized 1D carbyne chains and the recently reported *sp*+*sp*^3^-hybridized 3D diamondyne, and they are all dynamically stable. Moreover, 3D-phenylacetylene is metallic and 3D-phenyldiacetylene is semiconductor with an indirect band gap of 0.58 eV, in contrast to the semimetallic nature of graphite. These results provide new insights for the development of novel carbon allotropes.

## Results and Discussion

**Figure 1:**
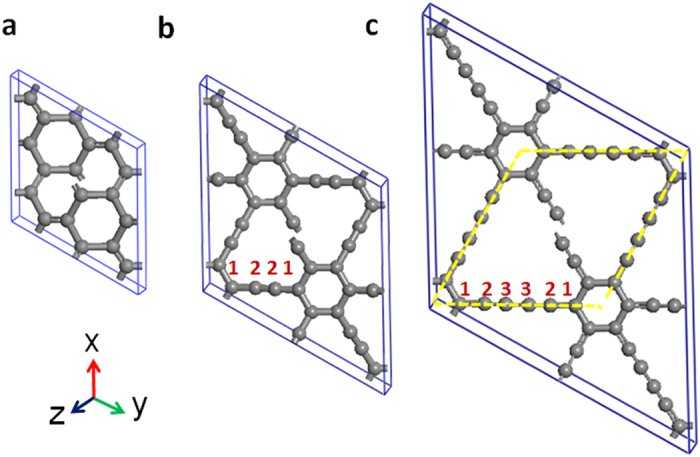
Three-dimensional carbon networks of rh6 polybenzene and polybenzene-ynes with phenylic rings and acetylenic chains in 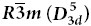 symmetry. (**a**) Rh6 polybenzene in an all *sp*^2^ 3D bonding network with lattice parameters a = 6.9022 Å, c = 3.470 Å, occupying the 18 h (0.8805, 0.1195, 0.5576) position, which comprises three zigzag benzene rings as its building blocks. (**b**) Rh12 phenylacetylene with lattice parameters a = 11.6050 Å and c = 3.6443 Å. The 18h1 (0.8579, 0.9289, 0.4849) atoms form three benzene rings and 18h2 (0.8617, 0.7233, 0.6021) atoms form nine acetylenic yne-bonds located between the benzene rings. (**c**) Rh18 phenyldiacetylene with lattice parameters a = 16.0963 Å and c = 4.1987 Å. The atoms on 18h1 (0.1023, 0.0511, 0.5065) site form three benzene rings; The atoms on 18h2 (0.1008, 0.8992, 0.4507) and 18h3 (0.1435, 0.8565, 0.3755) sites form nine butadiyne located between the benzene rings. The primitive cell is marked by yellow lines.

We first present the structural characterization of the simplest 3D phenylacetylene network constructed by inserting triple (–C≡C–) yne-bonds into the rh6 polybenzene lattice (see Fig. 1a), as well as the so-called 2D graphyne by inserting triple yne-bonds into an expanded graphene sheet^21^. This new carbon phase has a 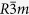 (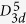) symmetry as that of rh6 polybenzene^3^ and topologically corresponds to 2D *γ*-graphyne^24^. In the hexagonal representation, it has a 36-atom hexagonal unit cell (see Fig. 1b) with lattice parameters *a* = 11.6050 Å, *c* = 3.6443 Å, occupying the 18h1 (0.8579, 0.9289, 0.4849) and 18h2 (0.8617, 0.7233, 0.6021) Wyckoff positions. The carbon atoms on the 18h1 sites form three benzene rings, as in rh6 carbon, with aromatic *sp*^2^ hybridization, while the carbon atoms on the 18h2 sites form nine triple yne-bonds located between the benzene rings with an ethyne-type *sp*-hybridization. As in rh6 carbon^3^, this structure also can be regarded as a three-dimensional chiral crystalline modification of carbyne connected via zigzag benzene rings with alternating single, triple and aromatic carbon-carbon bonds. It contains three distinct carbon-carbon bond lengths, a longer bond of 1.433 Å (C_1_–C_1_) in the benzene rings and two shorter bonds of 1.389 Å (C_1_–C_2_) and 1.232 Å (C_2_–C_2_) associated with the single and triple bond in carbyne chains, respectively. Meanwhile, there are three different bond angles, 171.13° for 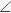
C_1_–C_2_–C_2_ along the carbyne chains, 119.39° for 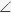
C_1_–C_1_–C_1_ inside and 120.21° for 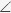
C_1_–C_1_–C_2_ out of the zigzag benzene rings, respectively. On the other hand, in the rhombohedral representation, it has a 12-atom primitive unit cell with equilibrium lattice parameters *a* = 6.8094 Å, *α* = 116.889°, occupying the 6 h (0.3428, 0.5560, 0.5560) and 6 h (0.4638, 0.4638, 0.8788) position, thus this 3D-phenylacetylene is also termed as rh12 carbon.

The construction of 3D-phenylacetylene can also be applied to design a 3D-phenyldiacetylene network (see Fig. 1c) by inserting butadiyne (–C≡C–C≡C–) segments between the benzene rings in the rh6 polybenzene lattice. The resulting structure has a 54-atom hexagonal unit cell with an equilibrium lattice parameters *a* = 16.0963 Å, *c* = 4.1987 Å, occupying the 18h1 (0.1023, 0.0511, 0.5065), 18h2 (0.1008, 0.8992, 0.4507), and 18h3 (0.1435, 0.8565, 0.3755) Wyckoff positions. The carbon atoms on the 18h1 sites form three benzene rings, while the carbon atoms on the 18h2 and 18h3 sites form nine diyne-bonds. This 3D network structure has a 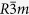 symmetry as in rh6 carbon, and topologically corresponds to 2D graphdiyne^24^, thus there are four distinct carbon-carbon bond lengths, a longer bond of 1.427 Å (C_1_–C_1_) is associated with the carbon atoms in the benzene rings and three shorter bonds of 1.395 Å (C_1_–C_2_), 1.233 Å (C_2_–C_3_), and 1.338 Å (C_3_–C_3_) are along the chains between the benzene rings. Note that the carbon chains are not perfectly linear with the bond angles of 172.54° for 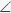
C_1_–C_2_–C_3_ and 180° for 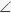
C_2_–C_3_–C_3_. In the rhombohedral representation, it has an 18-atom primitive unit cell, thus it also termed as rh18 carbon. The two new structures introduced here represent a new type of carbon allotropes that consist of phenyl rings connected by linear acetylenic chains in *sp*+*sp*^2^ bonding networks.

**Figure 2:**
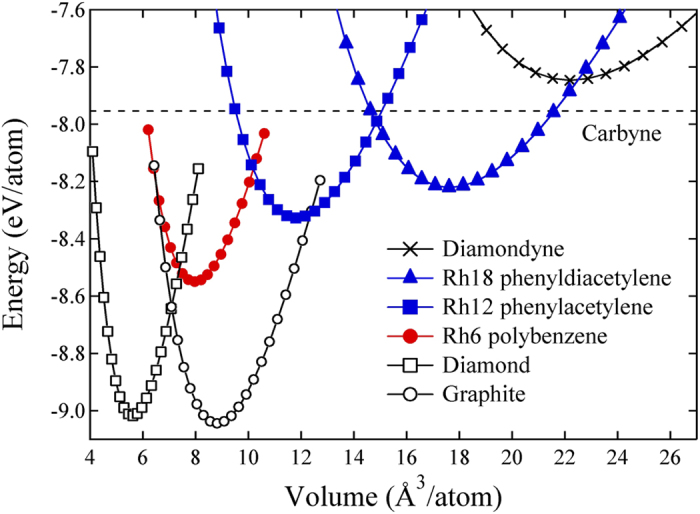
Energy versus volume per atom. Results for rh12 phenylacetylene and rh18 phenyldiacetylene compared to those of diamondyne, rh6 polybenzene, graphite and diamond. The dashed line indicates the energy level of 1D carbyne chain.

The total energies of rh12 phenylacetylene and rh18 phenyldiacetylene as a function of volume are shown in Fig. 2 in comparison with the results for diamond, graphite, rh6 carbon, carbyne, and diamondyne. Our calculated energetic data establish the stability sequence: diamondyne < carbyne < rh18 < rh12 < rh6. It is clearly seen that the rh12 and rh18 polybenzene-ynes are located between the energy range for rh6 carbon and carbyne, with an energy gain of about 0.07 eV per atom, while the diamondyne is out of the energy range, and even less favorable than carbyne.

By fitting the calculated total energy as a function of volume to Murnaghan’s equation of state^42^, we obtain the bulk modulus (*B*_0_) of rh12 and rh18 carbon as 195 and 129 GPa, respectively. The atomic densities are estimated to be 2.50, 1.69, and 1.14 g/cm^3^ for rh6, rh12, and rh18 carbon, respectively, which are considerably different from 3.51 g/cm^3^ for diamond and 2.27 g/cm^3^ for graphite. We can see that with increasing of the acetylenic chain length, the atomic density and bulk modulus are decreasing to a level even lower than those of graphite. The calculated equilibrium structural parameters, total energy, and bulk modulus for diamond, rh6 polybenzene, rh12-phenylacetylene, rh18-phenyldiacetylene, and graphite are listed in Table 1 and compared to available experimental data^43^.

**Figure 3:**
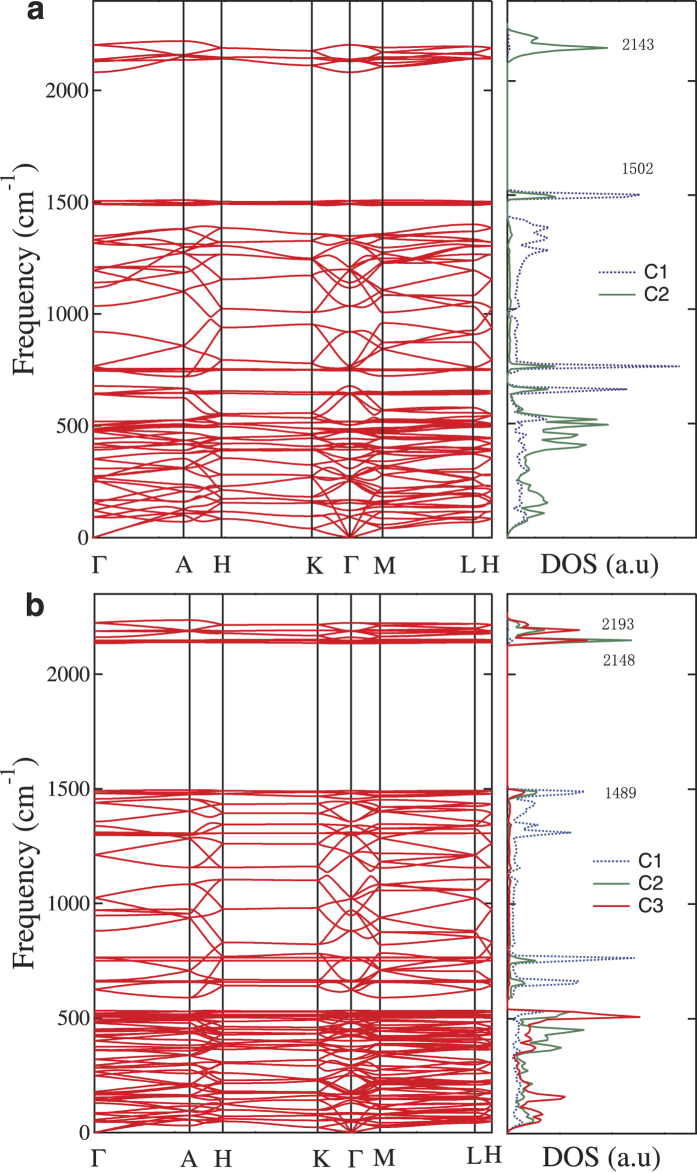
Phonon band structures and partial density of states (DOS). Results for rh12 phenylacetylene (**a**) and rh18 phenyldiacetylene (**b**). The spectra due to the triple bonds and phenyl bonds occur around 2150 cm^−1^ and 1500 cm^−1^, respectively. A clearer picture for the low vibrational modes is given in Fig. S1 in Supplementary Information.

We next examine the dynamic stability of the 3D *sp*+*sp*^2^ bonding networks by phonon mode analysis. Figure 3a shows the phonon band structures and density of state (DOS) for the 3D phenylacetylene. The obtained phonon eigenvalues can be explained well by considering the bonding nature of the phenyl and triple carbon-carbon bonds. The vibrational modes due to the triple yne-bond can be observed clearly around 2150 cm^−1^ with the carbon-carbon bond length of 1.232 Å (C_2_–C_2_) and the vibrational modes due to the phenyl bonds are distributed around 1500 cm^−1^ with the carbon-carbon bond length of 1.433 Å (C_1_–C_1_). The combination modes of phenyl and triple bonds of carbon atoms can be seen clearly below 730 cm^−1^. No imaginary frequencies were observed throughout the entire phonon band structures, thus confirming the dynamic stability of the 3D-phenylacetylene. Meanwhile, there is a large phonon band gap in the frequency range of 1500 and 2100 cm^−1^. Similar dynamic stability and vibrational modes are also confirmed for 3D phenyldiacetylene as shown in Fig. 3b. However, in the latter case there are two yne-modes around 2148 and 2193 cm^−1^ due to the diyne bonds related to the C_2_ and C_3_ carbon atoms.

To further examine the thermal stability, we have also performed *ab initio* molecular dynamics simulations using a 1 × 1 × 2 hexagonal supercell, which contains 6 primitive cells. After being heated at room temperature (300 K) and 1000 K for 3 ps with a time step of 1 fs, no structural changes occurred for both rh12 and rh18 carbon. These results show that rh12 and rh18 carbon are viable carbon allotrope for experimental synthesis.

**Figure 4:**
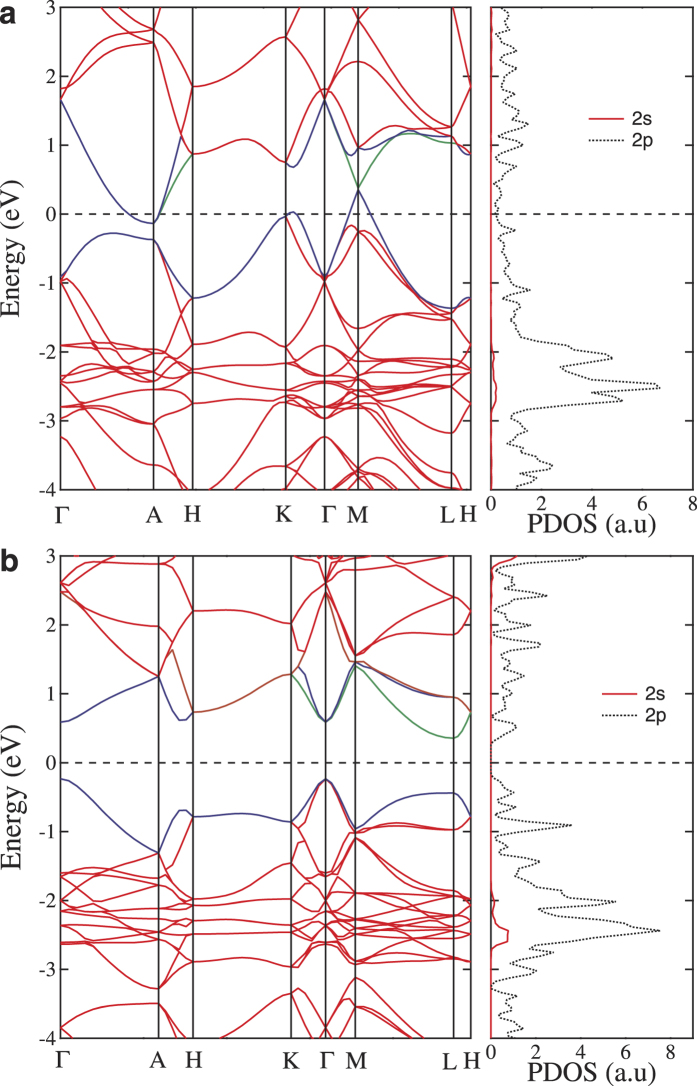
Electronic band structures and projected density of states (PDOS). Results for rh12 phenylacetylene (**a**) and rh18 phenyldiacetylene (**b**). The Fermi level is set at zero eV as indicated by the dashed lines.

Figure 4 shows the calculated electronic band structures and projected density of states (PDOS) by using the hybrid functionals (HSE06)^39^. The calculated band gap for diamond is about 5.36 eV, which is closed to the experimental data of 5.47 eV^43^, indicating the validity of the HSE06 method in predicting the band gaps for diamond and related *sp*^3^ bonded carbon structures. For 3D-phenylacetylene, as shown in Fig. 4a, there are three bands around the A, K and M points across the Fermi level, resulting in the metallic nature of the system. Meanwhile, for 3D-phenyldiacetylene, as shown in Fig. 4b, the conduction band minimum and valence band maximum are located at the L and Γ point, respectively, showing a semiconductor character with an indirect band gap of 0.58 eV. Moreover, from the PDOS, we can see that the states around the Fermi level are mainly coming from the 2*p* orbitals, which define the metallic nature for phenylacetylene and the band gap for phenyldiacetylene.

**Figure 5:**
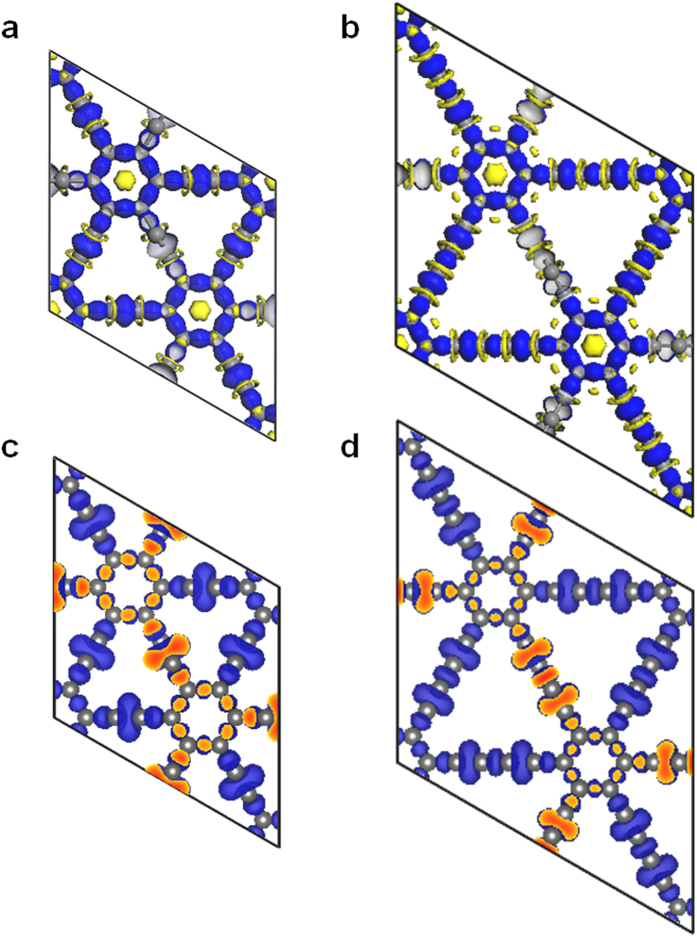
Electron density difference and electron localization function maps. (**a**,**b**) The electron density difference (EDD) for rh12 carbon (**a**) and rh18 carbon (**b**) with an isodensity of 0.1 e/Å^3^. The blue colour denotes a gain and the yellow colour a loss of electron density. (**c**,**d**) The electron localization function (ELF) for rh12 carbon (**c**) and rh18 carbon (**d**) with an isosurface level of 0.75.

To understand the bonding nature of electrons in both rh12 and rh18 carbon, the electron density difference (EDD) and electron localization function (ELF) maps are illustrated in Fig. 5. The EDD maps represent the variation of electron density in terms of chemical bonding. One can make an EDD plot by subtracting the overlapping atomic electron density from the self-consistent electron density of a crystal. The ELF maps can give a clear and quantitative description on the basic chemical bond (high ELF values 1 > ELF > 0.5 indicate the formation of covalent bonds)^44^,^45^,^46^, which was initially proposed by Becke and Edgecombe^45^ based on Hartree-Fock theory, and generalized by Savin *et al*.^46^ based on density functional theory. From the EDD maps shown in Fig. 5a,b, we can be seen that there is a larger gain of electron density between triple bonded carbons. Meanwhile, from the ELF maps shown in Fig. 5c,d, we can see that the electrons are well localized along the carbon chain, and the enhanced localization between triple bonded carbons can be seen clearly, which is more than the localization between the single and aromatic bonded carbons. These results show the strong triple bonding nature in the *sp*+*sp*^2^ hybrid networks. Furthermore, to understand the aromaticity in the phenyl rings, the nuclear-independent chemical shift NICS(0) at the center of the phenyl rings are calculated using the gauge-including atomic orbital (GIAO) method at the B3LYP/6-311 + G(d,p) level^47^. The NICS(0) values are estimated to be −8.16 ppm for benzene, −5.23 ppm for rh12 carbon, and −6.57 ppm for rh18 carbon. These results show that the aromaticity in phenyl rings of rh12 and rh18 carbon are smaller than the aromaticity in benzene.

**Figure 6:**
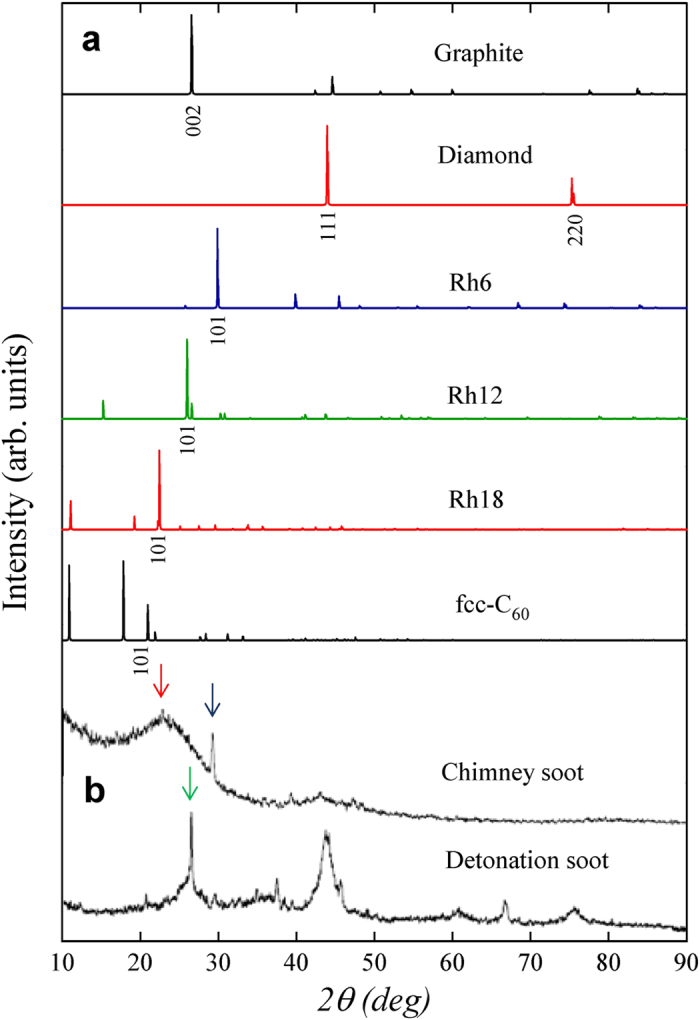
X-ray diffraction (XRD) patterns. (**a**) Simulated XRD patterns for graphite, diamond, rh6 polybenzene, rh12 phenylacetylene, rh18 phenyldiacetylene, and fcc C_60_. (**b**) Experimental XRD patterns for chimney soot and detonation soot^48^. The black, green, and red arrows indicate the XRD peaks corresponding to the main peak for rh6, rh12, and rh18 carbon, respectively. X-ray wavelength is 1.5406 Å with a copper source.

Finally, we plot in Fig. 6a simulated x-ray diffraction (XRD) patterns for graphite, diamond, rh6 polybenzene, rh12 phenylacetylene, rh18 phenyldiacetylene and and fcc C_60_, compared to the experimental data for chimney soot and detonation soot^48^. It is shown that the main (101) peak at 30° for rh6 should shift to 26° for rh12 and 22.4° for rh18 carbon. The main (101) peak at 26° for rh12 is very close to the main (002) peak at 26.5° for graphite, thus rh12 carbon may coexist with graphite in the detonation soot (see Fig. 6b and the Fig. 2 in ref. 48). On the other hand, for chimney soot, as shown in Fig. 6b, there is a sharp peak at 29.8°, which is fit well by the rh6 (101) diffraction peak, while a broad diffraction around 23°, which matches the rh18 (101) diffraction peak. These results suggest that rh12 and rh18 carbon as well as rh6 carbon may be present in chimney soot, carbon black or detonation soot^48^.

## Conclusions

In conclusion, we have identified by *ab initio* calculations a new type of three-dimensional carbon allotropes that consist of phenyl rings connected by linear acetylenic chains in *sp*+*sp*^2^ bonding networks. The resulting 3D phenylacetylene and phenyldiacetylene network structures in 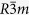 (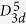) symmetry are topologically corresponding to 2D graphyne and graphdiyne, respectively, and they are energetically more favorable than the *sp*-hybridized 1D carbyne chains and the recently reported *sp*+*sp*^3^-hybridized 3D diamondyne. Phonon calculations show that these newly predicted structures are all dynamically stable. Electronic band and density of states calculations indicate that 3D-phenylacetylene with acetylenic yne-bonds is metallic, while 3D-phenyldiacetylene with diacetylenic yne-bonds is a semiconductor with an indirect band gap of 0.58 eV. Moreover, a detailed XRD analysis shows that phenylacetylene and phenyldiacetylene 3D network structures as well as rh6 polybenzene match the experimental diffraction peaks seen in the carbon black, diesel soot or chimney soot^48^. Our findings suggest a novel strategy in constructing carbon framework structures that may help solve the structures of the newly discovered but unidentified carbon phases seen in recent detonation experiments.

## Methods

Our calculations are carried out using the density functional theory as implemented in the Vienna *ab initio* simulation package (VASP)^36^. The generalized gradient approximation (GGA) developed by Armiento-Mattsson (AM05)^37^ were adopted for the exchange-correlation potential. The all-electron projector augmented wave (PAW) method^38^ was adopted with 2*s*^2^2*p*^2^ treated as valence electrons. A plane-wave basis set with a large energy cutoff of 800 eV was used. Forces on the ions are calculated through the Hellmann-Feynman theorem allowing a full geometry optimization. The energy minimization is done over the atomic and electronic degrees of freedom using the conjugate gradient iterative technique. Convergence criteria employed for both the electronic self-consistent relaxation and the ionic relaxation were set to 10^−8^ eV and 0.01 eV/Å for energy and force, respectively. A hybrid density functional method based on the Heyd-Scuseria-Ernzerhof scheme (HSE06)^39^ has been used to calculate electronic properties. Phonon calculations are performed using the phonopy code^40^ based on the supercell approach^41^ with a (1 × 1 × 2) 72-atom hexagonal supercell for rh12 and a (1 × 1 × 2) 108-atom hexagonal supercell for rh18 carbon. *Ab initio* molecular dynamics simulations under constant temperature (300 K and 1000 K) and volume (NVT) were performed to check thermal stability with a time step of 1 fs up to 3 ps. The nuclear-independent chemical shift NICS(0) at the center of the phenyl rings are calculated using the gauge-including atomic orbital (GIAO) method at the B3LYP/6-311 + G(d,p) level^47^ as as implemented in Gaussian03.

## Additional Information

**How to cite this article**: Wang, J.-T. *et al*. Three-Dimensional Carbon Allotropes Comprising Phenyl Rings and Acetylenic Chains in *sp+sp*^2^ Hybrid Networks. *Sci. Rep*. **6**, 24665; doi: 10.1038/srep24665 (2016).

## Supplementary Material

Supplementary Information

## Figures and Tables

**Table 1:** Calculated equilibrium structural parameters (volume *V*_0_, lattice parameters *a* and *c*, bond lengths *d*_*C*−*C*_), total energy *E*_*tot*_, bulk modulus *B*_0_, and electronic band gap *E*_*g*_ for rh6 polybenzene, rh12 phenylacetylene, rh18 phenyldiacetylene, graphite, and diamond at zero pressure, compared to available experimental data^43^.

Structure	Method	*V*_0_(Å^3^/atom)	a (Å)	c (Å)	*d*_*C*−*C*_ (Å)	*E*_*tot*_ (eV)	*B*_0_ (GPa)	*E*_*g*_ (eV)
Diamond	LDA-LAA	5.604	3.552		1.538	−9.018	451	5.36
	Exp^43^	5.673	3.567		1.544		446	5.47
Rh6 polybenzene	LDA-LAA	7.968	6.9022	3.470	1.359, 1.483	−8.550	299	0.47
Rh12 phenylacetylene	LDA-LAA	11.81	11.605	3.644	1.232–1.433	−8.327	195	
Rh18 phenyldiacetylene	LDA-LAA	17.45	16.096	4.199	1.233–1.427	−8.221	129	0.58
Graphite	LDA-LAA	8.813	2.462	6.710	1.422	−9.045	280	
	Exp^43^	8.783	2.460	6.704	1.420		286	
