# Supplementary material for: Three-Dimensional Carbon Allotropes Comprising Phenyl Rings and Acetylenic Chains in sp+sp2 Hybrid Networks
Source: Sci Rep. 2016 Apr 18;6:24665. doi: 10.1038/srep24665 (PMC4834540; doi:10.1038/srep24665)
Supplement: Supplementary Information [file srep24665-s1.doc]

**Supplementary information for “Three-Dimensional Carbon Allotropes Comprising Phenyl Rings and Acetylenic Chains in sp+sp^2^ Hybrid Networks”**

Jian-Tao Wang^1,*^, Changfeng Chen^2^, Han-Dong Li^3^, Hiroshi Mizuseki^4^ & Yoshiyuki Kawazoe^5,6^

*^1^Beijing National Laboratory for Condensed Matter Physics, Institute of Physics, Chinese Academy of Sciences, Beijing 100190, China*

*^2^Department of Physics and High Pressure Science and Engineering Center, University of Nevada, Las Vegas, Nevada 89154, USA*

*^3^State Key Laboratory of Environmental Criteria and Risk Assessment, Chinese Research Academy of Environmental Sciences, Beijing 100012, China*

*^4^Computational Science Research Center, Korea Institute of Science and Technology (KIST), Hwarangno 14-gil 5, Seongbuk-gu, Seoul 02792, Republic of Korea*

*^5^New Industry Creation Hatchery Center, Tohoku University, Sendai 980-8579, Japan*

*^6^Institute of Thermophysics, Siberian Branch of Russian Academy of Sciences, Novosibirsk 630090, Russia.*

*Correspondence and requests for materials should be addressed to J.T.W. [wjt@aphy.iphy.ac.cn].

**Phonon band structures**

To describe the characters for the acoustic branches, the phonon band structures at low vibrational modes are shown in Fig. S1. It is clearly seen that rh18 carbon is indeed softer than rh12 carbon as indicated by their acoustic branches.

**Figure S1│**Phonon band structures for (a) rh12 carbon and (b) rh18 carbon. The lowest three acoustic branches in both structures are indicated by green, blue, and black lines.
